# The *Salmonella* type-3 secretion system-1 and flagellar motility influence the neutrophil respiratory burst

**DOI:** 10.1371/journal.pone.0203698

**Published:** 2018-09-11

**Authors:** Trina L. Westerman, Lydia Bogomolnaya, Helene L. Andrews-Polymenis, M. Katherine Sheats, Johanna R. Elfenbein

**Affiliations:** 1 Department of Clinical Sciences, College of Veterinary Medicine, North Carolina State University, Raleigh, NC, United States of America; 2 Department of Microbial Pathogenesis and Immunology, College of Medicine, Texas A&M University, Bryan, TX, United States of America; 3 Institute of Fundamental Medicine and Biology, Kazan Federal University, Kazan, Russia; New York State Department of Health, UNITED STATES

## Abstract

Neutrophils are innate immune response cells designed to kill invading microorganisms. One of the mechanisms neutrophils use to kill bacteria is generation of damaging reactive oxygen species (ROS) via the respiratory burst. However, during enteric salmonellosis, neutrophil-derived ROS actually facilitates *Salmonella* expansion and survival in the gut. This seeming paradox led us to hypothesize that *Salmonella* may possess mechanisms to influence the neutrophil respiratory burst. In this work, we used an *in vitro Salmonella*-neutrophil co-culture model to examine the impact of enteric infection relevant virulence factors on the respiratory burst of human neutrophils. We report that neutrophils primed with granulocyte-macrophage colony stimulating factor and suspended in serum containing complement produce a robust respiratory burst when stimulated with viable STm. The magnitude of the respiratory burst increases when STm are grown under conditions to induce the expression of the type-3 secretion system-1. STm mutants lacking the type-3 secretion system-1 induce less neutrophil ROS than the virulent WT. In addition, we demonstrate that flagellar motility is a significant agonist of the neutrophil respiratory burst. Together our data demonstrate that both the type-3 secretion system-1 and flagellar motility, which are established virulence factors in enteric salmonellosis, also appear to directly influence the magnitude of the neutrophil respiratory burst in response to STm *in vitro*.

## Introduction

Non-typhoidal salmonellae are a leading cause of bacterial food-borne gastroenteritis with disease characterized by a marked neutrophilic intestinal inflammation [1, 2]. *Salmonella* invades non-phagocytic intestinal epithelial cells using the type-3 secretion system-1 (TTSS) encoded on *Salmonella* Pathogenicity Island-1 (SPI-1). The TTSS-1 secreted effector proteins are necessary for epithelial cell invasion, epithelial cell inflammatory signaling and neutrophil recruitment to the intestine [3–5]. Neutrophil recruitment is further enhanced by flagellin, which attracts neutrophils via activation of epithelial cell toll-like receptor 5 (TLR-5) [6]. Salmonellae survive neutrophilic infiltration in part through ROS detoxification by peroxidases and catalases and utilize the oxidizing conditions in the inflamed gut to gain advantage over resident microbes [7–9]. The multiple ways in which *Salmonella* interacts with neutrophils in the intestine are testimony to the integral role that the neutrophil inflammatory response plays in *Salmonella’s* survival strategy.

In order to recruit neutrophils to the *Salmonella*-infected epithelium, intestinal epithelial cells produce inflammatory mediators including granulocyte macrophage-colony stimulating factor (GM-CSF) and interleukin 8 (IL-8, CXCL8) [10–12]. Quiescent circulating neutrophils transition to a state of amplified responsiveness (“priming”) when exposed to inflammatory cytokines and bacterial products to prepare them for an amplified response to a second stimulus [13, 14]. One of the “primed” components of the neutrophil antimicrobial arsenal is the production of toxic reactive oxygen species (ROS) during the respiratory burst [15]. The functional capacity of the resulting neutrophil phenotype is ultimately directed by interaction with both host- and pathogen-derived molecules [13, 16]. For example, neutrophils respond to lipopolysaccharide decorating the surface of complement-coated *Salmonella* with a robust respiratory burst [17]. However, the ROS generated by neutrophils during enteritis is insufficient to kill *Salmonella* as it lives within luminal neutrophils during enteritis [18]. It is likely that *Salmonella* has evolved strategies to mitigate the severity of the neutrophil respiratory burst to promote its survival in the inflamed gut.

The purpose of our study was to establish and utilize an *in vitro* neutrophil-STm co-culture system to investigate the impact of *Salmonella* Typhimurium (STm) virulence factors important for intestinal infection on the respiratory burst of primary human neutrophils *in vitro*. We found that neutrophils produced a robust respiratory burst in response to STm when primed with GM-CSF in the presence of complement. Our data demonstrate that STm induced for the expression of the TTSS-1 elicit a robust neutrophil respiratory burst. Using deletion mutants, we demonstrate that both the TTSS-1 and found that flagellar motility are agonists for the neutrophil respiratory burst in response to STm. Overall, our data suggest that *Salmonella* virulence factors that play a key role during enterocolitis, the TTSS-1 and flagellar motility, influence the magnitude of the neutrophil respiratory burst in response to *Salmonella*.

## Materials and methods

### Bacterial strains and growth conditions

All bacterial strains were derivatives of *Salmonella* Typhimurium (STm) ATCC 14028.s and are listed in Table 1. Mutations were moved into a clean genetic background by P22 transduction and antibiotic cassettes were removed as previously described [19, 20]. Bacteria were grown on Luria Bertani (LB) agar or in LB broth at 37°C with agitation (250 rpm) unless otherwise noted. Media was supplemented with the following antibiotics as appropriate: nalidixic acid (50 mg/L), chloramphenicol (20 mg/L), kanamycin (50 mg/L), and carbenicillin (100 mg/L).

**Table 1:** Bacterial strains and plasmids.

**Strain**	**Genotype**	**Reference or Source**
HA420	ATCC14028.s (Spontaneous Nal-R)	Bogomolnaya 2008
JE598	HA420 ΔSPI-1::cm (Nal-R, Cm-R)	This study
JE524 JE526	14028 Δ*fliC*::kan Δ*fljB*::cm (Kan-R, Cm-R) 14028 Δ*fliC*::frt Δ*fljB*::frt ΔSPI-1::kan (Kan-R)	This study This study
JE13	14028 + pTurboGFP-B (Amp-R)	This study
JE1028	14028 ΔSPI-1::cm + pTurboGFP-B (Cm-R, Amp-R)	This study
JE1032	14028 Δ*fliC*::kan Δ*fljB*::cm + pTurboGFP-B (Kan-R, Cm-R, Amp-R)	This study
JE1296	14028 Δ*prgH*::kan + pTurboGFP-B (Kan-R, Amp-R)	This study
JE1298	14028 Δ*motA*::kan + pTurboGFP-B (Kan-R, Amp-R)	This study
JE1178	HA420 Δ*prgH*::kan (Nal-R, Kan-R)	This study
JE1204	JE1178 + pWSK29 (Nal-R, Kan-R, Amp-R)	This study
JE1207	JE1178 + pWSK29::*prgH* (Nal-R, Kan-R, Amp-R)	This study
JE1202	HA420 Δ*motA*::kan (Nal-R, Kan-R)	This study
JE1208	JE1202 + pWSK29 (Nal-R, Kan-R, Amp-R)	This study
JE1211	JE1202 + pWSK29::*motA* (Nal-R, Kan-R, Amp-R)	This study
JE239	HA420 + pNN387 (Nal-R, Cm-R)	Zheng 2013
JE240	HA420 + pNN387::rpsMp (Nal-R, Cm-R)	Zheng 2013
JE241	HA420 + pNN387::prgHp (Nal-R, Cm-R)	Zheng 2013
JE1290	JE1202 + pNN387 (Nal-R, Kan-R, Cm-R)	This study
JE1291	JE1202 + pNN387::rpsMp (Nal-R, Kan-R, Cm-R)	This study
JE1293	JE1202 + pNN387::prgHp (Nal-R, Kan-R, Cm-R)	This study
**Plasmid**	**Description**	**Reference or Source**
pTurboGFP-B	P_*lacO*_-TurboGFP; Amp-R	Evrogen
pCP20	flp recombinase; Amp-R	Datsenko 2000
pWSK29	Cloning vector; Amp-R	Wang 1991
pWSK29::*prgH*	pWSK29::*prgH*; Amp-R	This study
pWSK29::*motA*	pWSK29::*motA*; Amp-R	This study

For neutrophil-STm co-culture, bacteria were prepared from overnight cultures of bacteria in stationary phase unless otherwise indicated. For induction of SPI-1 gene expression, STm was grown in invasion-inducing conditions [21]. Bacteria were grown to late exponential phase by diluting overnight cultures 1:100 into LB broth and incubating at 37°C with agitation for 3 hours. Bacterial cultures were washed in phosphate buffered saline (PBS) and cell density was estimated by optical density (600 nm). Bacteria were diluted in PBS and maintained on ice until use. Where indicated, bacteria were killed by the addition of 10% formalin for 10 minutes. Diluted bacteria were plated to establish the number of viable colony forming units.

### Complementing plasmid construction

PCR products of *prgH* and *motA* were generated by colony PCR using Q5 polymerase (New England Biolabs). The PCR reaction for *prgH* was performed using an annealing temperature of 63°C with an extension time of 40s for 35 cycles. Restriction sites for endonucleases were incorporated into the primer sequences to facilitate cloning. A 1.8kb product for *prgH* was generated with the following primers:

prgHEcoRIFwd 5’ GTCGAATTCGTGGCCATTGACCTCTTCAAG 3’ and prgHHindIIIRev 5’ GTCAAGCTTCAAATTTTGCTGAGACGTCATCC 3’. The PCR reaction for *motA* was performed using an annealing temperature of 65°C with an extension time of 40s for 35 cycles. The 1.5kb product for *motA* was obtained with the following primers: motABamH1Fwd 5’ GTCGGATCCAAGGGATGCTGCCATTTTCG 3’ and motAKpn1Rev 5’ GTCGGTACCTCGGCGTAGGCAATTTTCCA 3’. The expected size of each of the PCR products was confirmed by agarose gel electrophoresis. The PCR product for *prgH* was digested with EcoRI and HindIII (New England Biolabs) and the *motA* product was digested with BamHI and KpnI (New England Biolabs) and purified with Qiaquick PCR purification kit (Qiagen). The inserts were cloned into pWSK29 [22] digested with the same endonucleases previously stated for each insert. Ligations were performed overnight at 14°C with T4 DNA ligase (New England Biolabs). Resulting constructs were transformed into chemically competent DH5α *Escherichia coli* using heat shock. Transformants were obtained by selection on LB agar with carbenicillin and X-gal (5-bromo-4-chloro-3-indolyl-β-D-galactopyranoside 40 μg/mL). Plasmids were isolated with Qiagen Miniprep kit (Qiagen), and correct insert size was confirmed by restriction digestion of plasmids, followed by confirmation of desired sequence by sequencing (Eton Bioscience). Complementing plasmids were transformed into electrocompetent restriction-deficient modification positive *S*. Typhimurium LB5000 by electroporation and transformants were isolated by selection on LB agar with carbenicillin [23]. Plasmids were isolated as above and then transformed into the electrocompetent *ΔprgH* and *ΔmotA* mutants by electroporation. Mutants bearing complementing plasmids were purified by streaking twice for single colonies on LB with carbenicillin and stored in glycerol stocks at -80°C.

### Human subjects

Human neutrophils were isolated from peripheral blood of healthy, adult volunteers. All participants provided written informed consent. This study was approved by the Institutional Research Ethics Committee of North Carolina State University (IRB approval #616).

### Neutrophil isolation and priming

Human neutrophils (PMN) were isolated from whole blood by Ficoll gradient centrifugation technique as previously described [24]. Briefly, dextran-sedimented (Sigma-Aldrich) leukocyte rich plasma was layered on sterile, endotoxin-free Ficoll-Paque solution (GE Healthcare) and centrifuged at 600g for 20 minutes. Red blood cells were removed by hypotonic NaCl lysis and the remaining neutrophils were washed once with Hank’s Buffered Saline Solution (HBSS) and suspended to a final concentration of 1x10^6^/ml in RPMI-1640 (with L-glutamine, without phenol red; Gibco) with 1mM Ca^++^, 1mM Mg^++^, and 10% normal human serum (NHS) from male AB donors (Corning). Where indicated, human serum was heat inactivated by incubation at 56°C for 30 minutes. Neutrophils isolated by this method routinely demonstrated greater than 98% viability as determined by trypan blue exclusion and greater than 95% purity.

Unless otherwise stated, purified neutrophils were primed with human recombinant granulocyte-macrophage colony stimulating factor (GM-CSF at 30 ng/mL; Millipore) for 30 minutes at 37°C with 5% CO_2_ [25]. Where indicated, purified neutrophils were primed with IL-8 (100 ng/mL; Sigma-Aldrich) for 5 minutes at 37°C with 5% CO_2_ [26]. Each experiment was performed using cells from at least 3 different donors.

### Neutrophil ROS measurement

Intracellular ROS was measured by dihydrorhodamine-123 (DHR; Adipogen) fluorescence as previously described [27]. Neutrophils (1.15x10^5^ total neutrophils) were aliquoted onto black polystyrene 96-well plates previously coated with 5% fetal calf serum (FCS; Hyclone) in sterile PBS. Unless otherwise stated, neutrophils were suspended in 10% NHS and primed with GM-CSF for 30 minutes prior to stimulation. Neutrophils were allowed to settle for 10 minutes prior to addition of 10μM DHR and appropriate stimulus. Neutrophils were stimulated with STm (PBS-washed and diluted), phorbol 12-myristate 13-acetate (PMA; 50ng/mL; Sigma-Aldrich) as a positive control [28], or TLR agonists lipopolysaccharide (LPS; BioXtra) or flagellin (Adipogen) isolated from *Salmonella* Typhimurium. STm were added at a multiplicity of infection (MOI) of 50:1 unless otherwise indicated. Unstimulated neutrophils and bacteria without neutrophils were included in each experiment as negative controls. Plates were incubated at 37°C with 5% CO_2_. Fluorescence (485 nm excitation, 530 nm emission) was read prior to incubation and then every 30–60 minutes using a plate reader (Synergy HTX, BioTek).

Intracellular and extracellular ROS were measured by luminol (5-amino-2,3-dihydro-1,4-phthalazinedione; Sigma-Aldrich) enhanced chemiluminescence as previously described [29]. For all luminol assays, neutrophils were suspended in 10% NHS and primed with GM-CSF for 30 minutes prior to stimulation. Neutrophils (1.15x10^5^ total neutrophils) were aliquoted onto white polystyrene 96-well plates previously coated with 5% FCS in PBS. Neutrophils were allowed to settle for 10 minutes prior to addition of 1mM luminol. Baseline luminescence measurement was obtained (integration time 1s; Synergy HTX, BioTek) and then neutrophils were stimulated with STm (MOI 50:1) or N-formylmethionyl-leucyl-phenylalanine (fMLP; 1μM; Sigma-Aldrich) as a positive control [30]. The plate was incubated at 37°C and luminescence measured every 5 minutes for 90 minutes.

### Flow cytometry

For neutrophil-STm cell association assays, neutrophils primed with GM-CSF were placed in polypropylene test tubes and exposed to STm constitutively expressing green fluorescent protein. Co-cultures were incubated at 37°C with 5% CO_2_ for the times indicated. Following incubation, cells were fixed in 2% paraformaldehyde and placed on ice for 10 minutes. Cells were centrifuged at 160g for 8 minutes and re-suspended in PBS. Cells were analyzed the same day on a CytoFLEX flow cytometer equipped with a 488 nm laser using CytExpert software (Beckman Coulter). The neutrophil population was gated by a forward scatter versus side scatter plot to discriminate from cell debris. Cell singlets were included by gating based on forward scatter-area versus forward scatter-width. Neutrophils not exposed to STm were used as a negative control for GFP-negative cells (GFP excitation 488 nm, emission 525). Data from 10,000 events gated on singlet neutrophils were collected and the percentage of GFP-positive neutrophils was calculated.

### Bacterial motility assays

Swimming and swarming assays were performed as previously described [31]. Swimming and swarming motility was assayed on plates containing 0.3% Difco Bacto agar (LB Miller base 25g/L) and 0.6% Difco Bacto agar (LB Miller base 25g/L with 0.5% dextrose), respectively. Overnight cultures were grown at 37°C with agitation and cell concentration was normalized by optical density. Bacterial strains were spotted onto swimming (5 μL) and swarming (10 μL) plates and incubated at 37°C for 4 or 6 hours, respectively. The diameter of each colony was measured and compared to the wild-type organism on the same plate. Each assay was performed on three separate occasions in 4–5 replicates.

### ß-galactosidase assays

For induction of SPI-1 expression, cultures of bacterial cells bearing plasmid constructs were grown in LB with appropriate antibiotic overnight. Overnight cultures were diluted 1:100 into LB with antibiotic and incubated at 37°C with agitation for 3h. ß-galactosidase activity (Miller units) was determined from cell pellets using standard methodology and calculated by the following equation: 1000 x [OD_420_ –(1.75 x OD_550_)] / [time x volume x OD_600_] [32].

### Data analysis

For DHR-123 fluorescence experiments, the fold change in fluorescence (relative fluorescence units, RFU) was calculated by dividing the RFU at the indicated time by the RFU at time zero for a given blood donor. The fluorescence fold change caused by the mutant was normalized to the WT for each blood donor. For luminol experiments, the peak and time to peak luminescence (relative luminescence units, RLU) stimulated by each bacterial strain were calculated. Statistical significance was determined using 2-way analysis of variance (ANOVA) with Tukey’s correction for multiple comparisons, or a one-way ANOVA with Dunnett’s correction for multiple comparisons, where indicated. Significance was set at *P*<0.05. Analyses were performed using GraphPad Prism version 7.0.

## Results

### Neutrophil priming and serum complement influence the STm-induced respiratory burst

**Fig 1:**
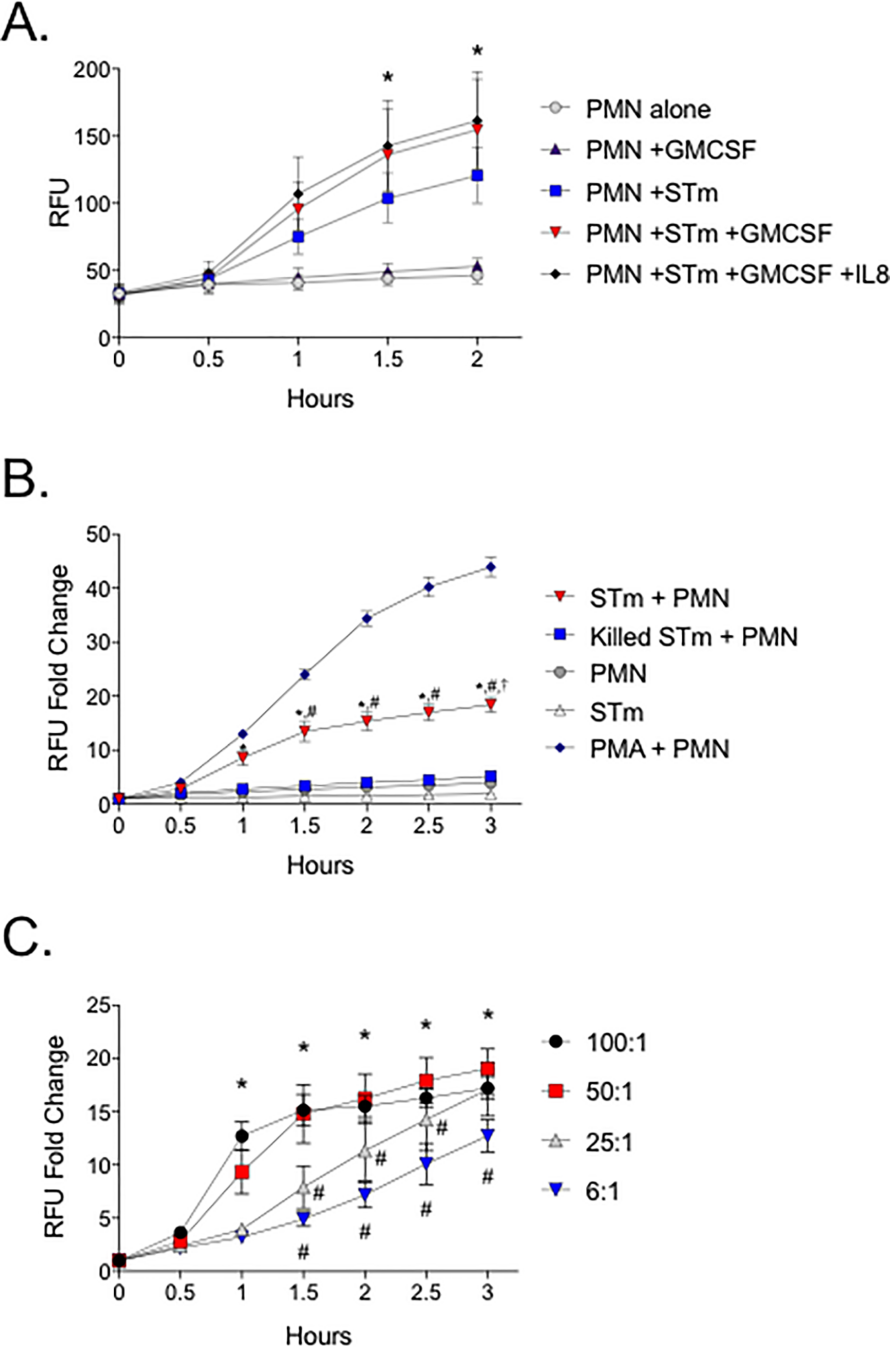
GM-CSF-primed human neutrophils produce a robust intracellular respiratory burst when stimulated with viable STm. (A) PMNs in NHS were either unprimed or primed with GM-CSF +/- IL-8 and stimulated with STm (MOI 50:1). * indicates difference from PMN+STm by two-way ANOVA with P<0.05. (B) GM-CSF-primed PMN in NHS were stimulated with viable or formalin-killed STm (MOI 50:1). * indicates difference from 0.5h, # from 1h and † from 2.5h by two-way ANOVA with P<0.05. (C) GM-CSF primed PMN in NHS were stimulated with STm at the indicated MOI. * indicates difference from 0.5h for MOI 50:1, # indicates difference from MOI 50:1 by two-way ANOVA with P<0.05. For all panels, data points indicate the mean +/- SEM from triplicate samples using blood from 3 different donors. Intracellular respiratory burst was assessed by DHR-123 fluorescence.

In order to select *in vitro* conditions that model neutrophil-STm interactions *in vivo* we first assessed the effect of different priming agents and serum components on the STm-induced intracellular respiratory burst. Both GM-CSF and IL-8 are chemoattractants and neutrophil priming agents released by the STm-infected epithelium [10–12]. When primed with GM-CSF, STm-stimulated neutrophils elicited a more robust intracellular respiratory burst than unprimed STm-stimulated neutrophils (Fig 1A). However, additional priming with IL-8 had no effect on the STm-induced intracellular respiratory burst; therefore IL-8 was not used as a priming agent for subsequent experiments. Next, we examined the effect of serum complement on the GM-CSF-primed neutrophils as complement is required for ingestion of STm in unprimed neutrophils [17]. We found that NHS, containing complement, increased the magnitude of the STm-induced intracellular neutrophil respiratory burst in GM-CSF-primed neutrophils (S1 Fig). These data suggest that a robust *in vitro* intracellular respiratory burst in response to STm occurs in the presence of complement and when neutrophils are primed with GM-CSF.

Next, we evaluated the kinetics of STm-stimulated intracellular neutrophil respiratory burst as assayed by DHR fluorescence. We observed significant intracellular ROS production at 1 hour and a further increase at 3 hours co-culture (Fig 1B). The kinetics of STm- and PMA-induced intracellular neutrophil respiratory were similar, but the PMA-induced respiratory burst was of a significantly greater magnitude (Fig 1B). To determine the contribution of pathogen-associated molecular patterns on the surface of STm to the STm-induced neutrophil respiratory burst, we exposed neutrophils to either formalin-killed whole pathogen or purified lipopolysaccharide (LPS) or flagellins. We found that neutrophils exposed to formalin-killed pathogen did not produce significant intracellular ROS (Fig 1B). In addition, we found no significant induction of intracellular neutrophil ROS from either purified LPS, flagellins, or a combination of the two (S2 Fig). Together these data suggest that STm viability has a significant impact on the intracellular respiratory burst in neutrophils *in vitro*.

Finally, in order to determine the effect of STm to neutrophil ratio on neutrophil intracellular ROS production in our *in vitro* model, we exposed neutrophils to increasing STm MOIs. We found a robust intracellular ROS production after 1 hour of co-culture at an MOI of 50:1, with no significant difference as the MOI increased to 100:1 (Fig 1C). The intracellular respiratory burst elicited by MOI of 25:1 and 13:1 (data not shown) was significantly lower than for 50:1 until 3 hours of co-culture (Fig 1C). The intracellular respiratory burst elicited by an MOI of 6:1 or less was significantly lower than 50:1 for 3 hours (Fig 1C). Guided by previous evidence established using macrophages [33], hypothesized that the effects of MOI on the DHR-measured respiratory burst was due to differences in numbers of STm-neutrophil interactions. To test our hypothesis, we exposed neutrophils to varied MOIs of GFP-expressing STm and used flow cytometry to establish the number of GFP-positive neutrophils. We found that there were significantly more GFP-positive neutrophils at an STm to neutrophil MOI of 50:1 as compared with 6:1 (Parts A and B in S3 Fig). The number of GFP-positive neutrophils in the high MOI increased from 30 minutes to 1 hour at which point it reached saturation, but the number of GFP-positive neutrophils exposed to the low MOI never reached saturation. These data suggest that the *in vitro* intracellular neutrophil respiratory burst as assessed by DHR fluorescence is increased when nearly all neutrophils interact with STm.

### Bacterial growth conditions alter the neutrophil respiratory burst

**Fig 2:**
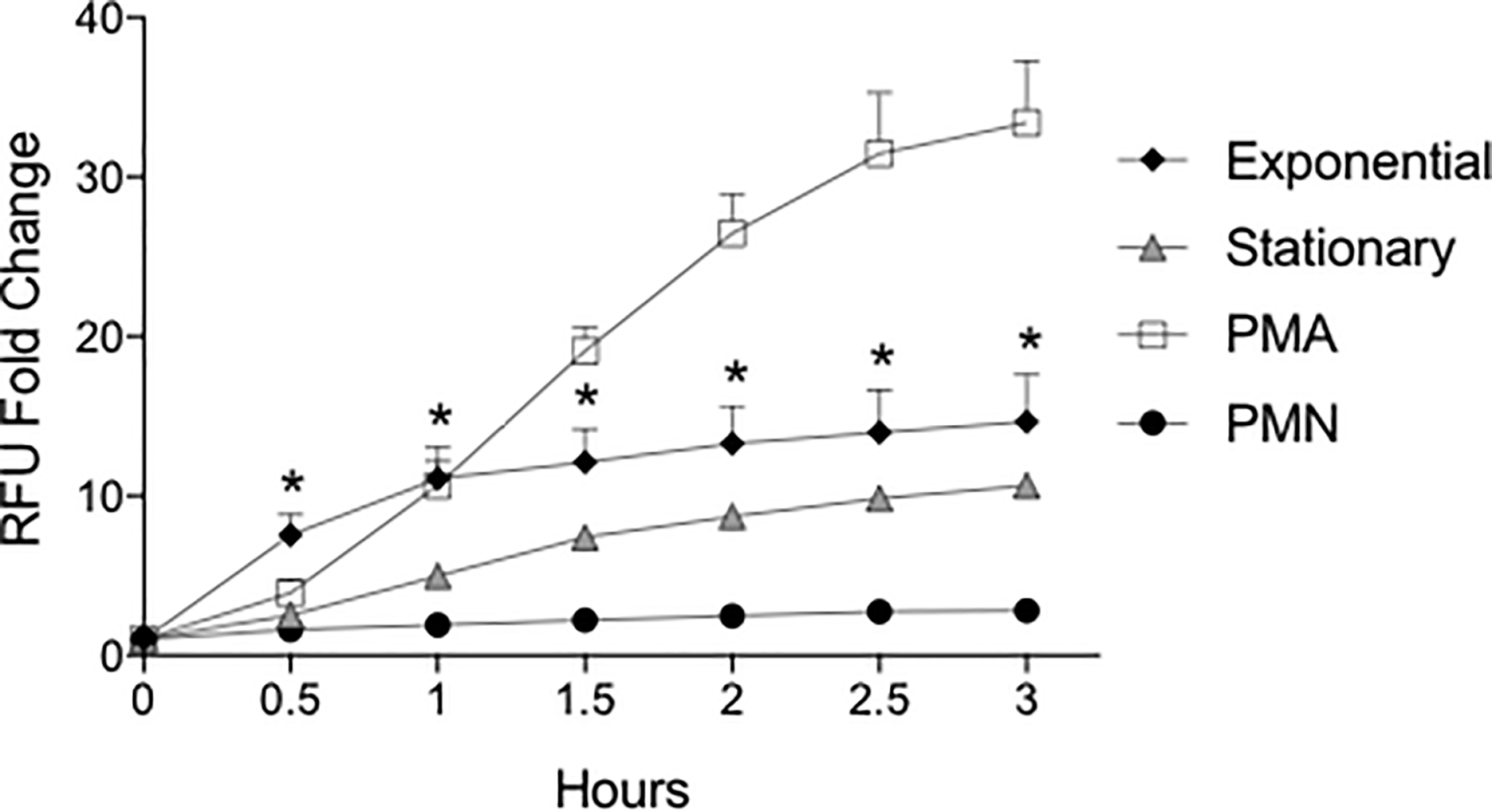
The neutrophil respiratory burst increases in response to STm induced for SPI-1 expression. GM-CSF-primed neutrophils in NHS were exposed to STm (MOI 50:1) from cultures in stationary phase or late-exponential phase. Respiratory burst was measured by DHR-123 fluorescence. Data points indicate the mean +/- SEM from triplicate samples using blood from 3 different donors. * indicates significant difference from stationary phase by two-way ANOVA with Bonferonni’s correction for multiple comparisons with *P*<0.05.

STm gene expression is altered during different growth phases *in vitro*. In order to increase STm invasion of epithelial cells *in vitro*, STm is cultured to aerobic late-exponential phase prior to co-culture with epithelial cells [21]. Bacteria prepared in this fashion have greater expression of invasion and flagellar genes as compared with microaerophilic stationary phase bacteria [21]. We therefore hypothesized that neutrophil ROS production in response to STm from late-exponential phase growth would be greater than the response to stationary phase bacteria. To test our hypothesis, we exposed neutrophils to STm from either stationary phase or late-exponential phase growth. We found a significant increase in the intracellular respiratory burst in neutrophils stimulated with STm grown to late-exponential phase as compared with stationary phase beginning at 30 minutes of co-culture (Fig 2). The neutrophil respiratory burst elicited by late-exponential phase STm was 3 times greater than that elicited by stationary phase STm at 30 minutes co-culture. These data suggest that variation in STm gene expression due to growth phase *in vitro* can alter the degree of neutrophil responses to STm.

### The TTSS-1 and motility induce the neutrophil respiratory burst

**Fig 3:**
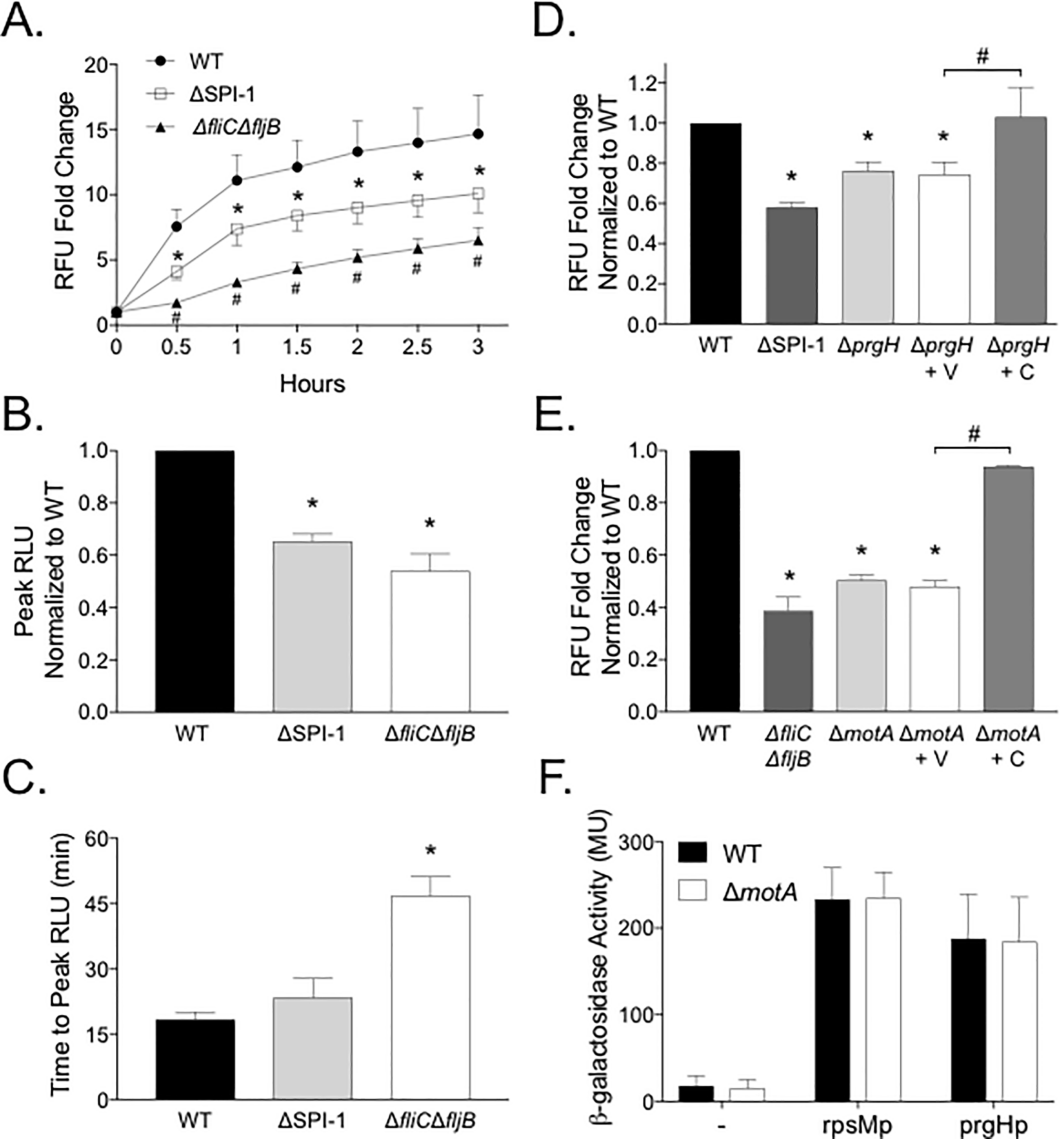
Both the STm TTSS-1 and flagellar motility induce a robust neutrophil respiratory burst. GM-CSF-primed neutrophils in NHS were exposed to STm (MOI 50:1) from cultures in late-exponential phase. (A) Intracellular respiratory burst as measured by DHR-123 fluorescence elicited by the WT (HA420) or the ΔSPI-1 (JE598) or Δ*fliC*Δ*fljB* (JE524) mutants. * indicates significant difference between the ΔSPI-1 mutant and the WT and # indicates significant difference between the Δ*fliC*Δ*fljB* mutant as compared to both the WT and ΔSPI-1 mutant by two-way ANOVA with P<0.05. (B) Peak total and (C) time to peak respiratory burst from PMN stimulated with the bacterial strains from (A) as measured by luminol-enhanced chemiluminescence. (B) Bars represent mean +/- SEM peak luminescence (RLU) normalized to the WT. (C) Bars represent mean +/- SEM time to peak luminescence. (B,C) * indicates significant difference from WT by one-way ANOVA with P<0.05. (D) Intracellular neutrophil respiratory burst after 2h co-culture with the WT (HA420), ΔSPI-1 mutant (JE598) Δ*prgH* mutant (JE1178), Δ*prgH* bearing the empty plasmid (JE1204) or the complementing plasmid (JE1207). (E) Intracellular neutrophil respiratory burst after 2h co-culture with the WT (HA420), Δ*fliC*Δ*fljB* mutant (JE524; data included from panel A for comparison), Δ*motA* mutant (JE1202), Δ*motA* mutant bearing the empty plasmid (JE1208) or complementing plasmid (JE1211). (D,E) * indicates significant difference from the WT and # indicates significant difference between the indicated strains by one-way ANOVA with *P*<0.05. (F) Activation of a terminal SPI-1 promoter (*prgHp-lacZY*) as determined by ß-galactosidase activity. (A-E) Data points indicate mean +/- SEM from triplicate samples using blood from 3 different donors. (F) Bars represent mean +/- SEM from 3 independent experiments.

STm uses environmental cues to induce the expression of the TTSS-1 and chemotaxis to interact with the intestinal epithelium *in vivo*. Our data suggest that STm from late-exponential phase growth, with increased expression of TTSS-1 and flagellar genes, elicit an increased neutrophil respiratory burst *in vitro*. We hypothesized that elimination of one or more of the known *Salmonella* virulence factors would alter the STm-induced intracellular neutrophil respiratory burst. We exposed neutrophils to either the virulent wild type organism, a ΔSPI-1 mutant (deleted for the entire SPI-1 locus), or a Δ*fliC*Δ*fljB* mutant (aflagellated). We found that neutrophils exposed to the ΔSPI-1 and Δ*fliC*Δ*fljB* mutants produced significantly less intracellular ROS compared to neutrophils exposed to the virulent WT organism (Fig 3A). In addition, the Δ*fliC*Δ*fljB* mutant induced significantly less neutrophil ROS than the ΔSPI-1 mutant (Fig 3A). To confirm our observations, we used luminol-enhanced chemiluminescence to measure total intracellular and extracellular neutrophil ROS. We found that the peak intracellular and extracellular ROS was significantly reduced upon stimulation with both the ΔSPI-1 and the Δ*fliC*Δ*fljB* mutant (Fig 3B and S4 Fig) although the time to peak ROS was significantly delayed by only the Δ*fliC*Δ*fljB* mutant (Fig 3C and S4 Fig). The difference in ROS production between the WT and mutants was not explained by a difference in numbers of neutrophils interacting with the STm mutants, as we found no difference in neutrophil association between the mutants and the WT organism by flow cytometry (Table 2).

**Table 2:** Neutrophil association with STm mutants after 1 hour co-culture.

		% GFP positive (mean +/- SEM)	
Strain	Genotype	Stationary	Exponential
JE13	WT	96 +/- 1.5	88 +/- 4.1
JE1028	*ΔSPI-1*	95 +/- 2.1	82 +/- 2.4
JE1032	*ΔfliCΔfljB*	99 +/- 0.2	94 +/- 1.4
JE1296	*ΔprgH*	98 +/- 0.4	92 +/- 3.0
JE1298	*ΔmotA*	97 +/- 0.7	86 +/- 2.4

- No differences found between WT and mutant within a growth condition by one-way ANOVA.

Our ΔSPI-1 mutant was deleted for the entire SPI-1 locus, including the regulator HilD that participates in regulation of other virulence determinants such as flagellar biogenesis and SPI-2 [34, 35]. Therefore, to verify the impact of the TTSS-1 on the neutrophil respiratory burst, we stimulated neutrophils with the avirulent Δ*prgH* mutant, which is defective for TTSS-1 needle complex formation, effector secretion, and invasion of epithelial cells [36–38]. We observed that the Δ*prgH*-stimulated neutrophils produce significantly less intracellular ROS than neutrophils stimulated with the virulent WT; this was reversed by complementation *in trans* (Fig 3D). To evaluate whether the diminished neutrophil respiratory burst in response to the ΔSPI-1 mutant could be explained by altered motility, we assessed the ability of the ΔSPI-1 mutant to swim and swarm on semi-solid agar. We found no difference in either swimming or swarming motility for the ΔSPI-1 mutant as compared with the WT (Parts A and B in S5 Fig). These results suggest that the STm TTSS-1 promotes ROS production in primary human neutrophils *in vitro*.

Our data demonstrate that an aflagellated, amotile mutant elicits a reduced respiratory burst (Fig 3A–3C and Parts A and B in S5 Fig). Furthermore, we documented no stimulation of neutrophil ROS with purified flagellins (S2 Fig). Given these findings, we hypothesized that the neutrophil respiratory burst would be diminished in response to an amotile mutant. We stimulated neutrophils with the Δ*motA* mutant, which is amotile (Parts A and B in S5 Fig) but has intact flagella [31, 39]. We show that the Δ*motA* mutant elicits significantly less intracellular neutrophil ROS than the WT organism (Fig 3E). Complementation *in trans* completely restored the ability of the Δ*motA* mutant to elicit intracellular neutrophil ROS (Fig 3E) and to swim and swarm on semi-solid agar (Parts A and B in S5 Fig). This difference was not explained by altered expression of the TTSS-1 in the Δ*motA* mutant, as we found no difference in terminal SPI-1 gene expression in our Δ*motA* mutant as compared with the WT (Fig 3F). As motility genes are expressed throughout STm growth in rich media *in vitro*, we hypothesized that the mutant grown to stationary phase would also stimulate a defective respiratory burst. We found a significant reduction in the intracellular neutrophil respiratory burst when stimulated with stationary phase Δ*motA* as compared with the WT (S6 Fig). Taken together, these data demonstrate that flagellar motility is a significant agonist of the *Salmonella*-stimulated neutrophil respiratory burst.

## Discussion

A massive influx of neutrophils into the gastrointestinal tract is a hallmark of enteric salmonellosis and the oxidative environment generated by the neutrophil respiratory burst enhances *Salmonella* colonization of the intestine. With this work, we report on the *in vitro* conditions under which we interrogated the *Salmonella*-induced neutrophil respiratory burst. We demonstrate that STm expression of the TTSS-1 increases the magnitude of the neutrophil respiratory burst. Furthermore, flagellar motility is a critical agonist of the neutrophil respiratory burst in response to STm.

Numerous methods have been developed to quantify ROS generated during the respiratory burst [40]. We chose two different methods to measure neutrophil ROS upon STm stimulation: DHR and luminol assays. DHR is a cumulative marker of overall intracellular oxidant levels as it is oxidized by many different ROS, rather than a direct measurement of superoxide radical, the primary product of the NADPH oxidase system [41, 42]. In contrast, luminol-enhanced chemiluminescence detects both intracellular and extracellular superoxide anion and hydrogen peroxide in the presence of myeloperoxidase [43]. Both of these methods have been successfully used in plate-based assays to evaluate the neutrophil respiratory burst [27, 29]. Consistent with prior work [44], our data show that neutrophils produce significant intracellular and extracellular ROS following stimulation with STm as measured by both the DHR (intracellular) and luminol (intracellular and extracellular) assays.

We sought to establish *in vitro* co-culture conditions for primary human neutrophil-*Salmonella* interaction that would model conditions encountered by neutrophils during enteric infection. Consistent with prior work using non-pathogenic stimulants [45], our data demonstrate that neutrophil priming with GM-CSF maximizes the intracellular neutrophil respiratory burst in response to STm *in vitro*. However, we did not detect an augmented intracellular respiratory burst with the addition of IL-8 to GM-CSF. Similar to previous studies, we found that normal serum, containing complement proteins, is needed for the optimal intracellular neutrophil respiratory burst in response to STm in our model, likely due to an increased STm uptake by neutrophils [17, 46–49]. Finally, we demonstrate that increasing the STm:neutrophil ratio increases the neutrophil respiratory burst, and plateaus when most neutrophils contact STm. One limitation of our data is the lack of ability to discriminate between intracellular and extracellularly bound STm as well as the number of bacteria per neutrophil. However, our data are consistent with the finding that neutrophils have a more robust respiratory burst when exposed to increasing relative concentrations of some bacteria including *Streptococcus pneumoniae* [50] and *Escherichia coli* [51]. Together our data demonstrate that the *in vitro* intracellular respiratory burst in STm-stimulated neutrophils is enhanced by complement, neutrophil priming with GM-CSF, and when all neutrophils contact at least one STm.

Our data support a role for the STm TTSS-1 in stimulating the neutrophil respiratory burst (Fig 3). Interestingly, this finding is in contrast to the effects of the TTSS of other pathogens on the neutrophil respiratory burst. The TTSS secreted effector proteins ExoS and ExoT of *Pseudomonas aeruginosa* block the neutrophil respiratory burst through inhibition of NADPH oxidase assembly [52]. The neutrophil respiratory burst is also inhibited by the TTSS encoded by the pCD1 virulence plasmid in *Yersinia pestis* [53]. Similarly, STm expressing the TTSS-2 is found within neutrophils in systemic infection models, suggesting that it may utilize the TTSS-2 to alter intracellular ROS production through the inhibition of the assembly of the NADPH oxidase system as it does to survive within macrophages during systemic infection [54–56]. However, unlike *P*. *aeruginosa* and *Y*. *pestis*, STm benefits from the oxidative environment created by neutrophils as a part of its pathogenesis in the gut [7–9], so it is not surprising that STm employs different mechanisms to alter neutrophil antimicrobial functions based on the local tissue environment. One possible mechanism for the TTSS-1 to increase the neutrophil respiratory burst is through a direct interaction of one or more TTSS-1 effector protein(s) with the assembly of the neutrophil NADPH oxidase system. Additionally, defective flagellin translocation into the host cell cytosol by the TTSS-1 deficient mutants may also account for the reduced respiratory burst as the TTSS-1 translocates flagellin into the cytosol of macrophages [57]. One limitation to our study is that we do not know whether the altered neutrophil ROS induced by the TTSS-1 mutant changes the fitness of STm inside neutrophils. Further work is needed to elucidate the mechanism(s) for the effects of the TTSS effector proteins on the neutrophil respiratory burst.

Flagellar motility is critical for STm to contact the gut epithelium and helps to increase the efficiency of SPI-1 mediated cell invasion in both the bovine and murine intestine [58–61]. Epithelial cell recognition of flagellins by TLR-5 causes IL-8 secretion, leading to neutrophil recruitment to the gut [6]. Our data demonstrate a reduced total and intracellular neutrophil respiratory burst in response to the amotile Δ*fliC*Δ*fljB* and Δ*motA* mutants by both fluorescent and luminescent methods of ROS detection (Fig 3). This diminished respiratory burst cannot be explained by a reduced cell-cell interaction, as we did not detect a change in neutrophil association with the amotile mutants (Table 2). Neither is this difference explained by altered TLR-5 flagellar activation, as purified STm flagellins alone failed to stimulate an intracellular respiratory burst as detected by DHR in our model. This is in contrast to prior work that demonstrated significant extracellular superoxide production by human neutrophils stimulated with *P*. *aeruginosa* flagellin type a or b (1 μg/mL) using diogenes-enhanced chemiluminescence [62]. Diogenes-enhanced chemiluminescence detects smaller quantities of ROS than DHR but only detects extracellular ROS [63]. However, our data are consistent with the finding that neutrophil extracellular trap formation in response to *Pseudomonas aeruginosa* requires flagellar motility [62]. Flagellar motility of *P*. *aeruginosa* directly stimulates neutrophil and macrophage phagocytosis through activation of the PI3K/Akt pathway [64, 65]. Phagocytosis stimulates neutrophil NADPH oxidase assembly [66], so it is possible that the amotile mutants are able to associate with neutrophils but are not phagocytosed as efficiently, leading to our observed phenotype. Our data taken together with prior work [62, 65] suggest an important role for flagellar motility in neutrophil antimicrobial responses. Further work is needed to establish how STm motility influences the neutrophil respiratory burst.

*Salmonella* benefits from the TTSS-1 mediated neutrophilic inflammation in the gut during early infection. STm gene expression is heterogeneous in the intestinal lumen. The TTSS-1 is expressed in about half of STm during early infection within the intestinal lumen [67]. Similarly, *fliC* expression is heterogeneous with the highest number of *fliC*-expressing cells located at the epithelial surface [67, 68]. Adding to this body of work, our data suggest that expression of the TTSS-1 and flagellar motility are agonists of neutrophil ROS production. Since STm are found within luminal neutrophils [18], it is possible that STm variation in virulence gene expression in the intestinal lumen alters neutrophil ROS production and contributes to STm survival in the inflamed intestine.

## Supporting information

S1 Fig Both complement and neutrophil priming are necessary for a robust intracellular neutrophil respiratory burst upon STm stimulation. PMNs were suspended in media containing either heat-inactivated serum (HS) or normal serum (NS) and primed with GM-CSF (G) with or without IL-8. PMNs were stimulated with STm (MOI 50:1) for 1 hour. Respiratory burst was measured by DHR-123 fluorescence. * indicates significant difference in relative fluorescence units (RFU) compared with unprimed STm-stimulated PMN in HS. Bars represent the mean +/- SEM RFU from triplicate samples using blood from 3 different donors. Statistical significance was determined on samples normalized to time 0 by one-way ANOVA with *P*<0.05.
(TIFF)

S2 Fig Neither purified flagellin nor LPS elicit a strong intracellular neutrophil respiratory burst. GM-CSF-primed human neutrophils in NHS were exposed to flagellin, LPS, or STm for 3 hours at the indicated concentrations. Intracellular respiratory burst was measured by DHR-123 fluorescence. Bars indicate mean +/- SEM fluorescence fold change from time 0 from triplicate samples from 3 blood donors. * indicates significant difference in fluorescence fold change from unstimulated neutrophils. Statistical significance was determined by one-way ANOVA with *P*<0.05.
(TIFF)

S3 Fig Neutrophil-STm association increases as MOI increases. GM-CSF-primed neutrophils in NHS were exposed to STm constitutively expressing GFP at the indicated MOI. The number of GFP-positive neutrophils was determined by flow cytometry. (A) Representative histogram from 1-hour co-culture. (B) Quantification of the proportion of GFP positive neutrophils after co-culture at the indicated MOI. Bars represent mean +/- SEM GFP positive PMNs from 3 blood donors. * indicates significant difference between groups. Different symbols (#, †) indicate significant difference within a group. Statistical significance determined by two-way ANOVA with *P*<0.05.
(TIFF)

S4 Fig Total respiratory burst from each individual donor as assessed by luminol-enhanced chemiluminescence. GM-CSF-primed neutrophils in NHS were exposed to STm (MOI 50:1) from cultures in late-exponential phase. See Fig 3A for strains. Data points represent the mean +/- SEM for triplicate samples from each donor.
(TIFF)

S5 Fig Both the SPI-1 mutant and complemented Δ*motA* mutant have adequate swimming and swarming motility. Normalized overnight cultures were spotted onto swimming (A) and swarming (B) agar. Cell spread was measured at 4 and 6 hours post-inoculation, respectively. The diameter of cell spread of each mutant was compared with the WT on the same plate. Each assay was performed in replicates of 4–5 on 3 different occasions. Bars represent mean +/- SEM. * indicates significant difference between WT and the mutant and # indicates significant difference between the indicated mutants by one-way ANOVA with P<0.05.
(TIFF)

S6 Fig The amotile mutant elicits a reduced respiratory burst from both stationary and exponentially grown cultures. GM-CSF-primed neutrophils in NHS were exposed to STm (MOI 50:1) from cultures in stationary (black bars) or late-exponential (white bars) phase. * indicates significant difference from the WT in the same condition by one-way ANOVA with *P*<0.05.
(TIFF)

## References

[1] Kirk MD, Pires SM, Black RE, Caipo M, Crump JA, Devleesschauwer B, et al World Health Organization Estimates of the Global and Regional Disease Burden of 22 Foodborne Bacterial, Protozoal, and Viral Diseases, 2010: A Data Synthesis. PLoS Med. 2015;12(12):e1001921 10.1371/journal.pmed.1001921 26633831 PMC4668831

[2] Boyd JF. Pathology of the alimentary tract in Salmonella typhimurium food poisoning. Gut. 1985;26(9):935–44. 3896961 10.1136/gut.26.9.935 PMC1432849

[3] Lee CA, Silva M, Siber AM, Kelly AJ, Galyov E, McCormick BA. A secreted Salmonella protein induces a proinflammatory response in epithelial cells, which promotes neutrophil migration. Proceedings of the National Academy of Sciences of the United States of America. 2000;97(22):12283–8. 10.1073/pnas.97.22.12283 11050248 PMC17333

[4] Raffatellu M, Wilson RP, Chessa D, Andrews-Polymenis H, Tran QT, Lawhon S, et al SipA, SopA, SopB, SopD, and SopE2 Contribute to Salmonella enterica Serotype Typhimurium Invasion of Epithelial Cells. Infection and immunity. 2005;73(1):146–54. 10.1128/IAI.73.1.146-154.2005 15618149 PMC538951

[5] Zhang S, Santos RL, Tsolis RM, Stender S, Hardt W-D, Bäumler AJ, et al The Salmonella enterica Serotype Typhimurium Effector Proteins SipA, SopA, SopB, SopD, and SopE2 Act in Concert To Induce Diarrhea in Calves. Infect Immun. 2002;70(7):3843–55. 10.1128/IAI.70.7.3843-3855.2002 12065528 PMC128071

[6] Gewirtz AT, Navas TA, Lyons S, Godowski PJ, Madara JL. Cutting edge: bacterial flagellin activates basolaterally expressed TLR5 to induce epithelial proinflammatory gene expression. J Immunol. 2001;167(4):1882–5. 11489966 10.4049/jimmunol.167.4.1882

[7] Faber F, Tran L, Byndloss MX, Lopez CA, Velazquez EM, Kerrinnes T, et al Host-mediated sugar oxidation promotes post-antibiotic pathogen expansion. Nature. 2016;534(7609):697–9. 10.1038/nature18597 27309805 PMC4939260

[8] Winter SE, Thiennimitr P, Winter MG, Butler BP, Huseby DL, Crawford RW, et al Gut inflammation provides a respiratory electron acceptor for Salmonella. Nature. 2010;467(7314):426–9. 10.1038/nature09415 20864996 PMC2946174

[9] Diaz-Ochoa VE, Lam D, Lee CS, Klaus S, Behnsen J, Liu JZ, et al Salmonella Mitigates Oxidative Stress and Thrives in the Inflamed Gut by Evading Calprotectin-Mediated Manganese Sequestration. Cell Host Microbe. 2016;19(6):814–25. 10.1016/j.chom.2016.05.005 27281571 PMC4901528

[10] Volf J, Boyen F, Faldyna M, Pavlova B, Navratilova J, Rychlik I. Cytokine response of porcine cell lines to Salmonella enterica serovar typhimurium and its hilA and ssrA mutants. Zoonoses Public Health. 2007;54(8):286–93. 10.1111/j.1863-2378.2007.01064.x 17894638

[11] Jung HC, Eckmann L, Yang SK, Panja A, Fierer J, Morzycka-Wroblewska E, et al A distinct array of proinflammatory cytokines is expressed in human colon epithelial cells in response to bacterial invasion. J Clin Invest. 1995;95(1):55–65. 10.1172/JCI117676 7814646 PMC295369

[12] Green SP, Chuntharapai A, Curnutte JT. Interleukin-8 (IL-8), Melanoma Growth-stimulatory Activity, and Neutrophil-activating Peptide Selectively Mediate Priming of the Neutrophil NADPH Oxidase through the Type A or Type B IL-8 Receptor. Journal of Biological Chemistry. 1996;271(41):25400–5. 8810307 10.1074/jbc.271.41.25400

[13] Colotta F, Re F, Polentarutti N, Sozzani S, Mantovani A. Modulation of granulocyte survival and programmed cell death by cytokines and bacterial products. Blood. 1992;80(8):2012–20. 1382715

[14] Kolaczkowska E, Kubes P. Neutrophil recruitment and function in health and inflammation. Nat Rev Immunol. 2013;13(3):159–75. 10.1038/nri3399 23435331

[15] El-Benna J, Hurtado-Nedelec M, Marzaioli V, Marie JC, Gougerot-Pocidalo MA, Dang PM. Priming of the neutrophil respiratory burst: role in host defense and inflammation. Immunol Rev. 2016;273(1):180–93. 10.1111/imr.12447 27558335

[16] Elbim C, Bailly S, Chollet-Martin S, Hakim J, Gougerot-Pocidalo MA. Differential priming effects of proinflammatory cytokines on human neutrophil oxidative burst in response to bacterial N-formyl peptides. Infection and immunity. 1994;62(6):2195–201. 8188340 10.1128/iai.62.6.2195-2201.1994 PMC186497

[17] van Bruggen R, Zweers D, van Diepen A, van Dissel JT, Roos D, Verhoeven AJ, et al Complement receptor 3 and Toll-like receptor 4 act sequentially in uptake and intracellular killing of unopsonized Salmonella enterica serovar Typhimurium by human neutrophils. Infection and immunity. 2007;75(6):2655–60. 10.1128/IAI.01111-06 17353285 PMC1932891

[18] Loetscher Y, Wieser A, Lengefeld J, Kaiser P, Schubert S, Heikenwalder M, et al Salmonella transiently reside in luminal neutrophils in the inflamed gut. PLoS One. 2012;7(4):e34812 10.1371/journal.pone.0034812 22493718 PMC3321032

[19] Sternberg NL, Maurer R. Bacteriophage-mediated generalized transduction in Escherichia coli and Salmonella typhimurium In: Jeffrey HM, editor. Methods Enzymol. Volume 204: Academic Press; 1991 p. 18–43. 1943777 10.1016/0076-6879(91)04004-8

[20] Datsenko KA, Wanner BL. One-step inactivation of chromosomal genes in Escherichia coli K-12 using PCR products. Proc Natl Acad Sci U S A. 2000;97(12):6640–5. 10.1073/pnas.120163297 10829079 PMC18686

[21] Ibarra JA, Knodler LA, Sturdevant DE, Virtaneva K, Carmody AB, Fischer ER, et al Induction of Salmonella pathogenicity island 1 under different growth conditions can affect Salmonella–host cell interactions in vitro. Microbiology. 2010;156(4):1120–33. 20035008 10.1099/mic.0.032896-0 PMC2848694

[22] Wang RF, Kushner SR. Construction of versatile low-copy-number vectors for cloning, sequencing and gene expression in Escherichia coli. Gene. 1991;100:195–9. 2055470

[23] Bullas LR, Ryu JI. Salmonella typhimurium LT2 strains which are r- m+ for all three chromosomally located systems of DNA restriction and modification. Journal of bacteriology. 1983;156(1):471–4. 6352690 10.1128/jb.156.1.471-474.1983 PMC215113

[24] Gresham HD, Clement LT, Lehmeyer JE, Griffin FM Jr., Volanakis JE., Stimulation of human neutrophil Fc receptor-mediated phagocytosis by a low molecular weight cytokine. J Immunol. 1986;137(3):868–75. 3522738

[25] Dang PM, Dewas C, Gaudry M, Fay M, Pedruzzi E, Gougerot-Pocidalo MA, et al Priming of human neutrophil respiratory burst by granulocyte/macrophage colony-stimulating factor (GM-CSF) involves partial phosphorylation of p47(phox). J Biol Chem. 1999;274(29):20704–8. 10400704 10.1074/jbc.274.29.20704

[26] Yuo A, Kitagawa S, Kasahara T, Matsushima K, Saito M, Takaku F. Stimulation and priming of human neutrophils by interleukin-8: cooperation with tumor necrosis factor and colony-stimulating factors. Blood. 1991;78(10):2708–14. 1726709

[27] Sheats MK, Pescosolido KC, Hefner EM, Sung EJ, Adler KB, Jones SL. Myristoylated Alanine Rich C Kinase Substrate (MARCKS) is essential to beta2-integrin dependent responses of equine neutrophils. Vet Immunol Immunopathol. 2014;160(3–4):167–76. 10.1016/j.vetimm.2014.04.009 24857637 PMC4108539

[28] Vowells SJ, Sekhsaria S, Malech HL, Shalit M, Fleisher TA. Flow cytometric analysis of the granulocyte respiratory burst: a comparison study of fluorescent probes. J Immunol Methods. 1995;178(1):89–97. 7829869 10.1016/0022-1759(94)00247-t

[29] Martin EM, Till RL, Sheats MK, Jones SL. Misoprostol Inhibits Equine Neutrophil Adhesion, Migration, and Respiratory Burst in an In Vitro Model of Inflammation. Front Vet Sci. 2017;4:159 10.3389/fvets.2017.00159 29034248 PMC5626936

[30] Dahlgren C, Karlsson A. Respiratory burst in human neutrophils. J Immunol Methods. 1999;232(1–2):3–14. 10618505 10.1016/s0022-1759(99)00146-5

[31] Bogomolnaya LM, Aldrich L, Ragoza Y, Talamantes M, Andrews KD, McClelland M, et al Identification of novel factors involved in modulating motility of Salmonella enterica serotype typhimurium. PLoS One. 2014;9(11):e111513 10.1371/journal.pone.0111513 25369209 PMC4219756

[32] Miller JH. Experiments in molecular genetics Cold Spring Harbor, N.Y.: Cold Spring Harbor Laboratory; 1972. xvi, 466 p. p.

[33] Achouri S, Wright JA, Evans L, Macleod C, Fraser G, Cicuta P, et al The frequency and duration of Salmonella-macrophage adhesion events determines infection efficiency. Philos Trans R Soc Lond B Biol Sci. 2015;370(1661):20140033 10.1098/rstb.2014.0033 25533091 PMC4275903

[34] Bustamante VH, Martínez LC, Santana FJ, Knodler LA, Steele-Mortimer O, Puente JL. HilD-mediated transcriptional cross-talk between SPI-1 and SPI-2. Proceedings of the National Academy of Sciences. 2008;105(38):14591–6. 10.1073/pnas.0801205105 PMC2567235 18799744

[35] Singer HM, Kühne C, Deditius JA, Hughes KT, Erhardt M. The Salmonella Spi1 Virulence Regulatory Protein HilD Directly Activates Transcription of the Flagellar Master Operon flhDC. Journal of bacteriology. 2014;196(7):1448–57. 10.1128/JB.01438-13 24488311 PMC3993337

[36] Schraidt O, Lefebre MD, Brunner MJ, Schmied WH, Schmidt A, Radics J, et al Topology and Organization of the Salmonella typhimurium Type III Secretion Needle Complex Components. PLOS Pathogens. 2010;6(4):e1000824 10.1371/journal.ppat.1000824 20368966 PMC2848554

[37] Klein JR, Fahlen TF, Jones BD. Transcriptional Organization and Function of Invasion Genes within Salmonella enterica Serovar Typhimurium Pathogenicity Island 1, Including the prgH,prgI, prgJ, prgK, orgA,orgB, and orgC Genes. Infection and immunity. 2000;68(6):3368–76. 10816487 10.1128/iai.68.6.3368-3376.2000 PMC97603

[38] Behlau I, Miller SI. A PhoP-repressed gene promotes Salmonella typhimurium invasion of epithelial cells. J Bacteriol. 1993;175(14):4475–84. 8392513 10.1128/jb.175.14.4475-4484.1993 PMC204888

[39] Muramoto K, Macnab RM. Deletion analysis of MotA and MotB, components of the force-generating unit in the flagellar motor of Salmonella. Mol Microbiol. 1998;29(5):1191–202. 9767587 10.1046/j.1365-2958.1998.00998.x

[40] Maghzal GJ, Krause KH, Stocker R, Jaquet V. Detection of reactive oxygen species derived from the family of NOX NADPH oxidases. Free Radic Biol Med. 2012;53(10):1903–18. 10.1016/j.freeradbiomed.2012.09.002 22982596

[41] Dupre-Crochet S, Erard M, Nubetae O. ROS production in phagocytes: why, when, and where? J Leukoc Biol. 2013;94(4):657–70. 10.1189/jlb.1012544 23610146

[42] Wardman P. Fluorescent and luminescent probes for measurement of oxidative and nitrosative species in cells and tissues: progress, pitfalls, and prospects. Free Radic Biol Med. 2007;43(7):995–1022. 10.1016/j.freeradbiomed.2007.06.026 17761297

[43] Bedouhene S, Moulti-Mati F, Hurtado-Nedelec M, Dang PM, El-Benna J. Luminol-amplified chemiluminescence detects mainly superoxide anion produced by human neutrophils. Am J Blood Res. 2017;7(4):41–8. 28804681 PMC5545213

[44] Schurmann N, Forrer P, Casse O, Li J, Felmy B, Burgener AV, et al Myeloperoxidase targets oxidative host attacks to Salmonella and prevents collateral tissue damage. Nat Microbiol. 2017;2:16268 10.1038/nmicrobiol.2016.268 28112722

[45] Khwaja A, Carver JE, Linch DC. Interactions of granulocyte-macrophage colony-stimulating factor (CSF), granulocyte CSF, and tumor necrosis factor alpha in the priming of the neutrophil respiratory burst. Blood. 1992;79(3):745–53. 1370644

[46] Dunkelberger JR, Song WC. Complement and its role in innate and adaptive immune responses. Cell Res. 2010;20(1):34–50. 10.1038/cr.2009.139 20010915

[47] Gondwe EN, Molyneux ME, Goodall M, Graham SM, Mastroeni P, Drayson MT, et al Importance of antibody and complement for oxidative burst and killing of invasive nontyphoidal Salmonella by blood cells in Africans. Proc Natl Acad Sci U S A. 2010;107(7):3070–5. 10.1073/pnas.0910497107 20133627 PMC2840319

[48] Mishra M, Ressler A, Schlesinger LS, Wozniak DJ. Identification of OprF as a complement component C3 binding acceptor molecule on the surface of Pseudomonas aeruginosa. Infection and immunity. 2015;83(8):3006–14. 10.1128/IAI.00081-15 25964476 PMC4496607

[49] Mishra M, Byrd MS, Sergeant S, Azad AK, Parsek MR, McPhail L, et al Pseudomonas aeruginosa Psl polysaccharide reduces neutrophil phagocytosis and the oxidative response by limiting complement-mediated opsonization. Cell Microbiol. 2012;14(1):95–106. 10.1111/j.1462-5822.2011.01704.x 21951860 PMC4466118

[50] Barbuti G, Moschioni M, Fumarulo R, Censini S, Montemurro P. Streptococcus pneumoniae modulates the respiratory burst response in human neutrophils. FEMS immunology and medical microbiology. 2010;60(1):57–62. 10.1111/j.1574-695X.2010.00716.x 20618848

[51] Watson RW, Redmond HP, Wang JH, Condron C, Bouchier-Hayes D. Neutrophils undergo apoptosis following ingestion of Escherichia coli. J Immunol. 1996;156(10):3986–92. 8621940

[52] Vareechon C, Zmina SE, Karmakar M, Pearlman E, Rietsch A. Pseudomonas aeruginosa Effector ExoS Inhibits ROS Production in Human Neutrophils. Cell Host Microbe. 2017;21(5):611–8 e5. 10.1016/j.chom.2017.04.001 28494242 PMC5478421

[53] Spinner JL, Seo KS, O'Loughlin JL, Cundiff JA, Minnich SA, Bohach GA, et al Neutrophils are resistant to Yersinia YopJ/P-induced apoptosis and are protected from ROS-mediated cell death by the type III secretion system. PLoS One. 2010;5(2):e9279 10.1371/journal.pone.0009279 20174624 PMC2823771

[54] van der Heijden J, Bosman ES, Reynolds LA, Finlay BB. Direct measurement of oxidative and nitrosative stress dynamics in Salmonella inside macrophages. Proceedings of the National Academy of Sciences. 2015;112(2):560–5. 10.1073/pnas.1414569112 PMC4299202 25548165

[55] Vazquez-Torres A, Xu Y, Jones-Carson J, Holden DW, Lucia SM, Dinauer MC, et al Salmonella Pathogenicity Island 2-Dependent Evasion of the Phagocyte NADPH Oxidase. Science. 2000;287(5458):1655–8. 10698741 10.1126/science.287.5458.1655

[56] Geddes K, Cruz F, Heffron F. Analysis of cells targeted by Salmonella type III secretion in vivo. PLoS Pathog. 2007;3(12):e196 10.1371/journal.ppat.0030196 18159943 PMC2151088

[57] Sun YH, Rolan HG, Tsolis RM. Injection of flagellin into the host cell cytosol by Salmonella enterica serotype Typhimurium. J Biol Chem. 2007;282(47):33897–901. 10.1074/jbc.C700181200 17911114

[58] Winter SE, Thiennimitr P, Nuccio SP, Haneda T, Winter MG, Wilson RP, et al Contribution of flagellin pattern recognition to intestinal inflammation during Salmonella enterica serotype typhimurium infection. Infect Immun. 2009;77(5):1904–16. 10.1128/IAI.01341-08 19237529 PMC2681779

[59] Stecher B, Hapfelmeier S, Muller C, Kremer M, Stallmach T, Hardt WD. Flagella and chemotaxis are required for efficient induction of Salmonella enterica serovar Typhimurium colitis in streptomycin-pretreated mice. Infect Immun. 2004;72(7):4138–50. 10.1128/IAI.72.7.4138-4150.2004 15213159 PMC427403

[60] Schmitt CK, Ikeda JS, Darnell SC, Watson PR, Bispham J, Wallis TS, et al Absence of all components of the flagellar export and synthesis machinery differentially alters virulence of Salmonella enterica serovar Typhimurium in models of typhoid fever, survival in macrophages, tissue culture invasiveness, and calf enterocolitis. Infect Immun. 2001;69(9):5619–25. 10.1128/IAI.69.9.5619-5625.2001 11500437 PMC98677

[61] Horstmann JA, Zschieschang E, Truschel T, de Diego J, Lunelli M, Rohde M, et al Flagellin phase-dependent swimming on epithelial cell surfaces contributes to productive Salmonella gut colonisation. Cell Microbiol. 2017. 10.1111/cmi.12739 28295924

[62] Floyd M, Winn M, Cullen C, Sil P, Chassaing B, Yoo D-g, et al Swimming Motility Mediates the Formation of Neutrophil Extracellular Traps Induced by Flagellated Pseudomonas aeruginosa. PLOS Pathogens. 2016;12(11):e1005987 10.1371/journal.ppat.1005987 27855208 PMC5113990

[63] Yamazaki T, Kawai C, Yamauchi A, Kuribayashi F. A highly sensitive chemiluminescence assay for superoxide detection and chronic granulomatous disease diagnosis. Trop Med Health. 2011;39(2):41–5. 10.2149/tmh.2011-08 22028609 PMC3153160

[64] Lovewell RR, Hayes SM, O'Toole GA, Berwin B. Pseudomonas aeruginosa flagellar motility activates the phagocyte PI3K/Akt pathway to induce phagocytic engulfment. American journal of physiology Lung cellular and molecular physiology. 2014;306(7):L698–707. 10.1152/ajplung.00319.2013 24487390 PMC3962627

[65] Demirdjian S, Hopkins D, Sanchez H, Libre M, Gerber SA, Berwin B. PIP3 Induces Phagocytosis of Non-Motile Pseudomonas aeruginosa. Infection and immunity. 2018. 10.1128/IAI.00215-18 PMC6056877 29844235

[66] Nguyen GT, Green ER, Mecsas J. Neutrophils to the ROScue: Mechanisms of NADPH Oxidase Activation and Bacterial Resistance. Front Cell Infect Microbiol. 2017;7:373 10.3389/fcimb.2017.00373 28890882 PMC5574878

[67] Laughlin RC, Knodler LA, Barhoumi R, Payne HR, Wu J, Gomez G, et al Spatial segregation of virulence gene expression during acute enteric infection with Salmonella enterica serovar Typhimurium. mBio. 2014;5(1):e00946–13. 10.1128/mBio.00946-13 24496791 PMC3950517

[68] Stecher B, Barthel M, Schlumberger MC, Haberli L, Rabsch W, Kremer M, et al Motility allows S. Typhimurium to benefit from the mucosal defence. Cell Microbiol. 2008;10(5):1166–80. 10.1111/j.1462-5822.2008.01118.x 18241212

