# Supplementary figures and images for: The Salmonella type-3 secretion system-1 and flagellar motility influence the neutrophil respiratory burst
Source: PLoS One. 2018 Sep 11;13(9):e0203698. doi: 10.1371/journal.pone.0203698 (PMC6133356; doi:10.1371/journal.pone.0203698)

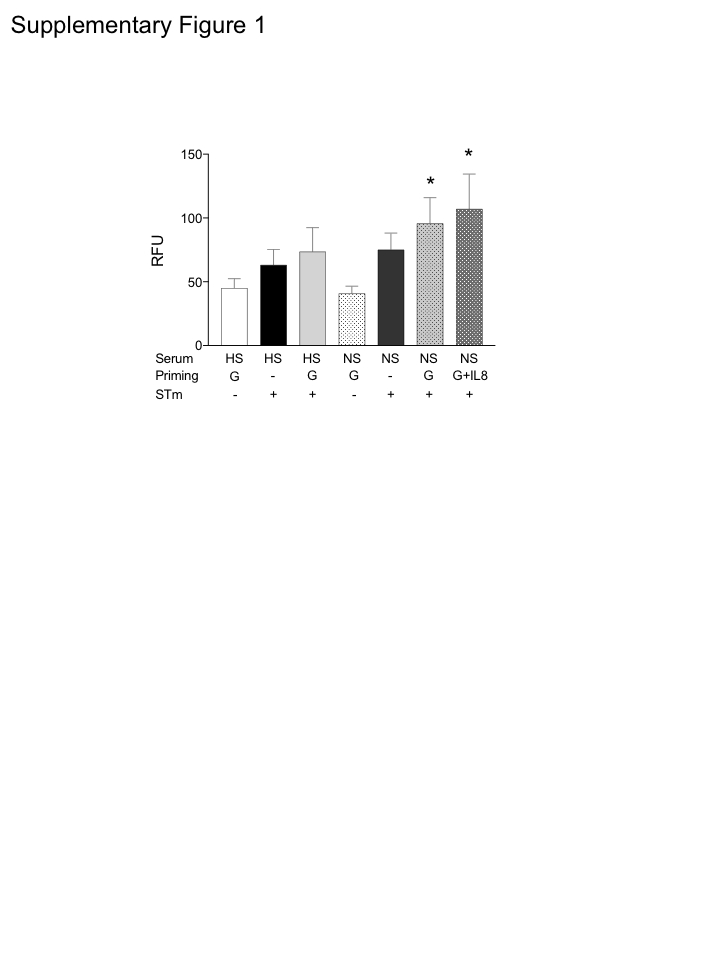

Supplement: S1 Fig — PMNs were suspended in media containing either heat-inactivated serum (HS) or normal serum (NS) and primed with GM-CSF (G) with or without IL-8. PMNs were stimulated with STm (MOI 50:1) for 1 hour. Respiratory burst was measured by DHR-123 fluorescence. * indicates significant difference in relative fluorescence units (RFU) compared with unprimed STm-stimulated PMN in HS. Bars represent the mean +/- SEM RFU from triplicate samples using blood from 3 different donors. Statistical significance was determined on samples normalized to time 0 by one-way ANOVA with P<0.05. (TIFF) [file pone.0203698.s001.tiff]

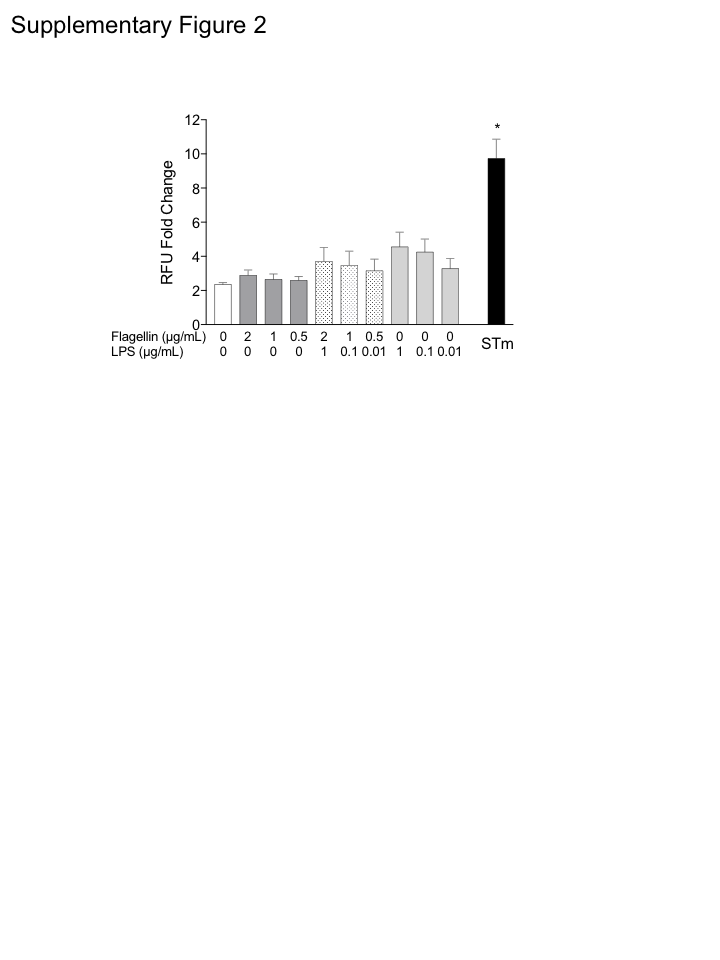

Supplement: S2 Fig — GM-CSF-primed human neutrophils in NHS were exposed to flagellin, LPS, or STm for 3 hours at the indicated concentrations. Intracellular respiratory burst was measured by DHR-123 fluorescence. Bars indicate mean +/- SEM fluorescence fold change from time 0 from triplicate samples from 3 blood donors. * indicates significant difference in fluorescence fold change from unstimulated neutrophils. Statistical significance was determined by one-way ANOVA with P<0.05. (TIFF) [file pone.0203698.s002.tiff]

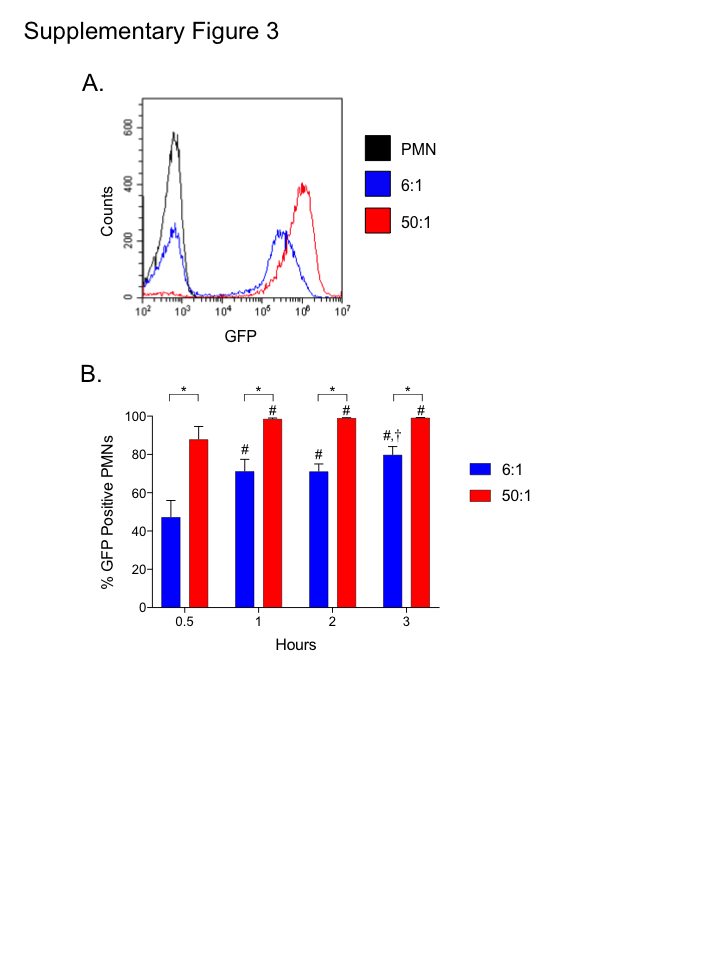

Supplement: S3 Fig — GM-CSF-primed neutrophils in NHS were exposed to STm constitutively expressing GFP at the indicated MOI. The number of GFP-positive neutrophils was determined by flow cytometry. (A) Representative histogram from 1-hour co-culture. (B) Quantification of the proportion of GFP positive neutrophils after co-culture at the indicated MOI. Bars represent mean +/- SEM GFP positive PMNs from 3 blood donors. * indicates significant difference between groups. Different symbols (#, †) indicate significant difference within a group. Statistical significance determined by two-way ANOVA with P<0.05. (TIFF) [file pone.0203698.s003.tiff]

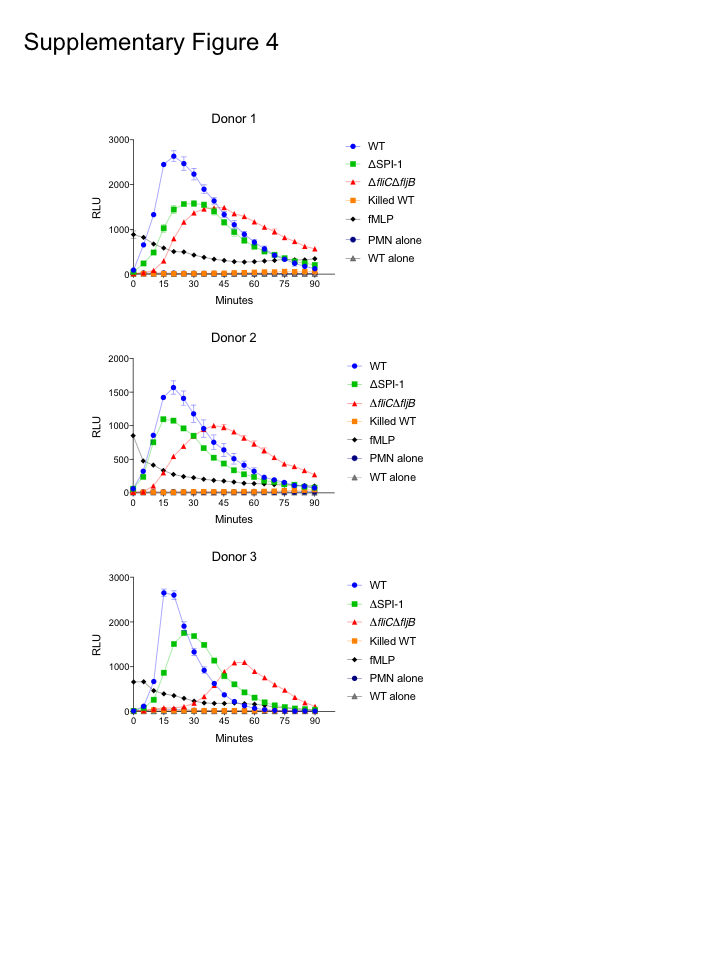

Supplement: S4 Fig — GM-CSF-primed neutrophils in NHS were exposed to STm (MOI 50:1) from cultures in late-exponential phase. See Fig 3A for strains. Data points represent the mean +/- SEM for triplicate samples from each donor. (TIFF) [file pone.0203698.s004.tiff]

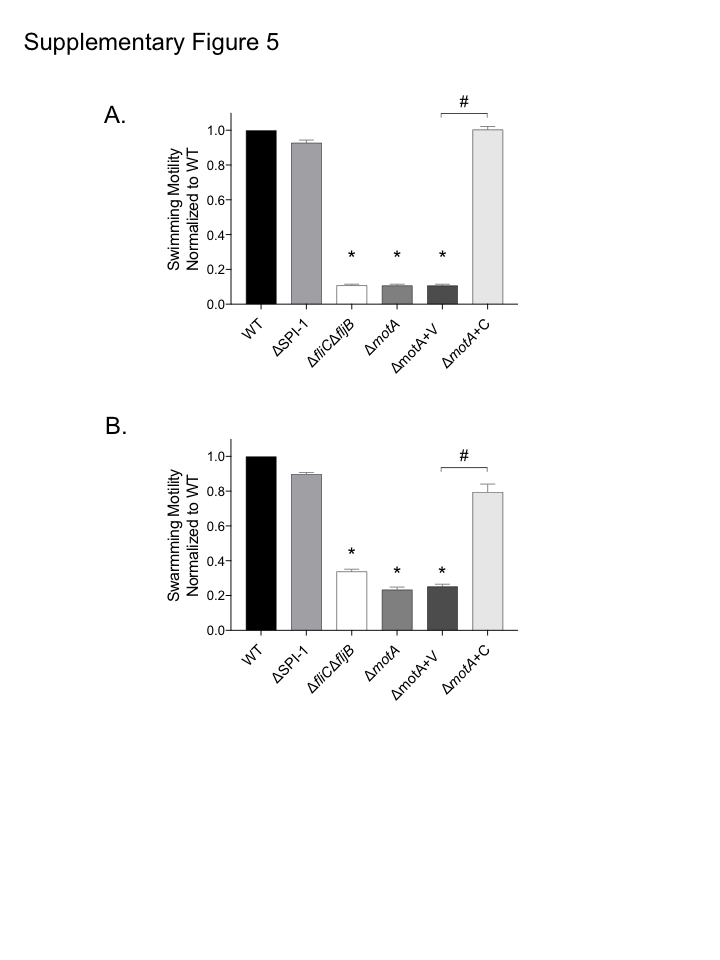

Supplement: S5 Fig — Normalized overnight cultures were spotted onto swimming (A) and swarming (B) agar. Cell spread was measured at 4 and 6 hours post-inoculation, respectively. The diameter of cell spread of each mutant was compared with the WT on the same plate. Each assay was performed in replicates of 4–5 on 3 different occasions. Bars represent mean +/- SEM. * indicates significant difference between WT and the mutant and # indicates significant difference between the indicated mutants by one-way ANOVA with P<0.05. (TIFF) [file pone.0203698.s005.tiff]

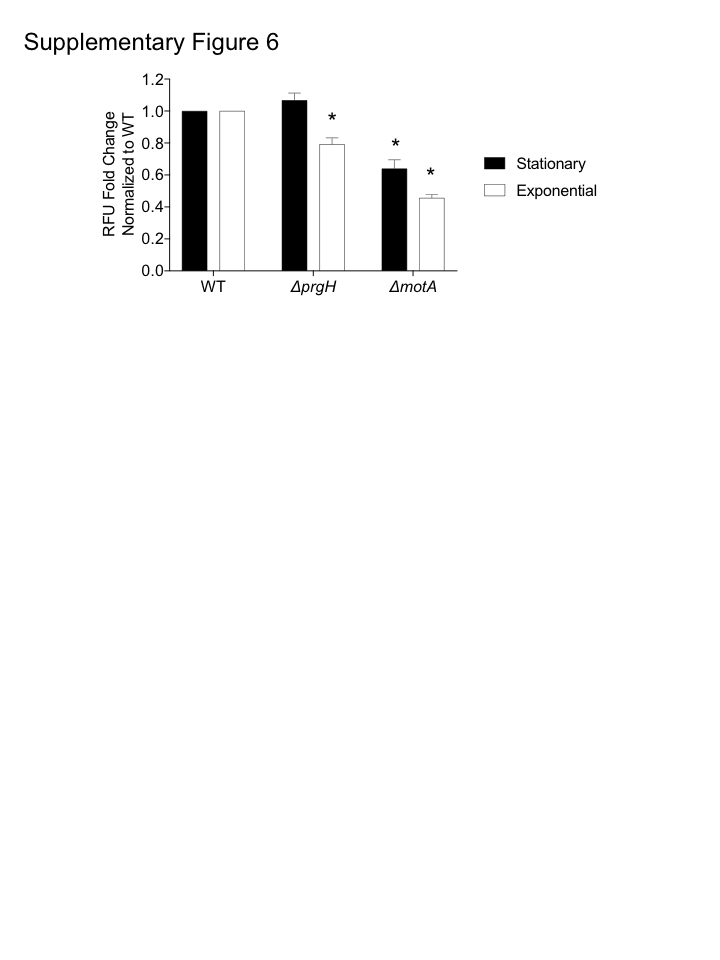

Supplement: S6 Fig — GM-CSF-primed neutrophils in NHS were exposed to STm (MOI 50:1) from cultures in stationary (black bars) or late-exponential (white bars) phase. * indicates significant difference from the WT in the same condition by one-way ANOVA with P<0.05. (TIFF) [file pone.0203698.s006.tiff]
